# Melanoma skin cancer detection using mask-RCNN with modified GRU model

**DOI:** 10.3389/fphys.2023.1324042

**Published:** 2024-01-16

**Authors:** K. M. Monica, J. Shreeharsha, Przemysław Falkowski-Gilski, Bozena Falkowska-Gilska, Mohan Awasthy, Rekha Phadke

**Affiliations:** ^1^ School of Computer Science and Engineering, Vellore Institute of Technology, Chennai, Tamil Nadu, India; ^2^ Department of Computer Science and Engineering, Rao Bahadur Y. Mahabaleswarappa Engineering College, Ballari, Karnataka, India; ^3^ Faculty of Electronics, Telecommunications and Informatics, Gdansk of Technology, Gdansk, Poland; ^4^ Specialist Diabetes Outpatient Clinic, Olsztyn, Poland; ^5^ Department of Engineering and Technology, Bharati Vidyapeeth Peeth Deemed to be University, Navi Mumbai, Maharashtra, India; ^6^ Department of Electronics and Communication Engineering, Nitte Meenakshi Institute of Technology, Bangalore, Karnataka, India

**Keywords:** faster region based convolutional neural network, gated recurrent unit, melanoma skin cancer detection, normalization, pre-trained models

## Abstract

**Introduction:** Melanoma Skin Cancer (MSC) is a type of cancer in the human body; therefore, early disease diagnosis is essential for reducing the mortality rate. However, dermoscopic image analysis poses challenges due to factors such as color illumination, light reflections, and the varying sizes and shapes of lesions. To overcome these challenges, an automated framework is proposed in this manuscript.

**Methods:** Initially, dermoscopic images are acquired from two online benchmark datasets: International Skin Imaging Collaboration (ISIC) 2020 and Human against Machine (HAM) 10000. Subsequently, a normalization technique is employed on the dermoscopic images to decrease noise impact, outliers, and variations in the pixels. Furthermore, cancerous regions in the pre-processed images are segmented utilizing the mask-faster Region based Convolutional Neural Network (RCNN) model. The mask-RCNN model offers precise pixellevel segmentation by accurately delineating object boundaries. From the partitioned cancerous regions, discriminative feature vectors are extracted by applying three pre-trained CNN models, namely ResNeXt101, Xception, and InceptionV3. These feature vectors are passed into the modified Gated Recurrent Unit (GRU) model for MSC classification. In the modified GRU model, a swish-Rectified Linear Unit (ReLU) activation function is incorporated that efficiently stabilizes the learning process with better convergence rate during training.

**Results and discussion:** The empirical investigation demonstrate that the modified GRU model attained an accuracy of 99.95% and 99.98% on the ISIC 2020 and HAM 10000 datasets, where the obtained results surpass the conventional detection models.

## 1 Introduction

In recent decades, skin cancer is one of the prevalent cancer types, which is categorized into two types such as non-melanoma and melanoma (Sreelatha et al., 2019; Ashraf et al., 2020). The accurate classification of different skin cancer types holds significant importance because it directly influences the choice of treatment to be pursued (Saba, 2021). Melanoma, scientifically referred to as malignant melanoma, is a type of cancer that originates from melanocytes. Data presented by the American cancer society indicates a consistent increase in melanoma rates over the past three decades (Abayomi-Alli et al., 2021). Although melanoma constitutes only around 1% of all skin cancer cases, it is responsible for a significant majority of skin cancer-related deaths (Babar et al., 2021). The most concerning aspect of melanoma is its capacity to extensively metastasize throughout the body via the lymphatic system and blood vessels (Wei et al., 2020). However, early detection translates to a high curability rate for melanoma. The conventional diagnostic procedure for melanoma involves a visual assessment conducted by a dermatologist, which is a time-consuming process and error prone (Mijwil, 2021; Thiyaneswaran et al., 2021).

Furthermore, there exist challenges when it comes to the detection of melanoma (Albahar, 2019). These difficulties encompass factors such as the morphology of individual lesions, the lighting conditions within the medical examination space, the patient’s skin color, and the expertise of the professional making the melanoma diagnosis (Divya and Ganeshbabu, 2020; Khan et al., 2021a; Priyadharshini et al., 2023). Currently, artificial intelligence is being continuously employed to aid physicians and dermatologists in the more efficient analysis of data, leading to enhanced accuracy and reliability in diagnoses across various domains (Mohakud and Dash, 2022a). Specifically, deep learning is implemented in skin cancer detection using diverse architectures, such as CNNs, Recursive Neural Network (RvNN), Recurrent Neural Network (RNN), etc. (Albahli et al., 2020; Cheong et al., 2021). Deep learning encounters four significant challenges in skin cancer detection: memory limitations, computational intensity, the vanishing gradient problem, and model complexity (Iyer et al.; Khan et al., 2021b). To address these challenges and attain accurate segmentation and classification of skin lesions, this manuscript introduces a novel deep learning-based automated framework.

The contributions are as follows:

• We implement a mask-RCNN model for partitioning cancerous regions in dermoscopic images acquired from the ISIC 2020 and HAM 10000 datasets. The mask-RCNN model efficiently segments and differentiates skin lesions, even in cases of high overlap between regions. The automated skin lesion segmentation by the mask-RCNN model significantly saves time for medical professionals and dermatologists.
• We integrate three pre-trained models (ResNeXt101, Xception, and InceptionV3) to extract relevant feature vectors from the partitioned regions. These three pre-trained CNN models capture texture features from higher-level objects and hierarchical features from lower-level edges. This hierarchy allows the modified GRU model to learn complex representations in dermoscopic images, resulting in high classification accuracy.
• We propose a modified GRU model to classify two types of skin lesions in the ISIC 2020 dataset and seven types of skin lesions in the HAM 10000 dataset. We use seven performance metrics to evaluate the proposed model’s efficacy, namely: Jaccard score, Dice score, Matthews Correlation Coefficient (MCC), accuracy, sensitivity, f1-score, and specificity.

The current manuscript is prepared in the following manner. Section 2 presents the literature survey, while Section 3 explains the mask-RCNN model, pre-trained models, and the modified GRU model. Sections 4, 5 provide the numerical results and the conclusion of this manuscript.

## 2 Literature survey

Thanh et al. (2020) developed an efficient framework for the automatic detection of MSC. An adaptive principal curvature technique was employed initially for detecting and removing hairs from dermoscopic images. Subsequently, a color normalization technique was applied to improve the visibility level of skin lesion regions for discriminating various skin tones. Finally, the Asymmetry-Border-Color-Diameter (ABCD) rule was utilized for effective MSC detection. Evaluation metrics, namely the Jaccard score, Dice score, and accuracy, confirmed the superiority of the developed framework in MSC detection. However, the ABCD rule was not sensitive enough in detecting MSC at an early stage. Additionally, not all melanomas adhere to the ABCD rule, some may exhibit irregular borders and asymmetry.

Nawaz et al. (2022) incorporated the Fuzzy K-Means clustering (FKM) technique with the RCNN model to achieve precise detection of MSC. Initially, the RCNN model was employed for enhancing visual information and removing noise from the collected dermoscopic images. Further, the FKM technique was applied for precisely segmenting the affected skin regions with variable boundaries and sizes. The developed RCNN-FKM model’s performance was assessed utilizing three benchmark datasets, and the results obtained clearly demonstrated that the RCNN-FKM model surpassed the performance of existing models. In this study, the FKM technique involves complex calculations related to the conventional k-means clustering technique, due to the introduction of membership degrees. Additionally, the FKM technique was more sensitive to noisy images, because it directly affects membership degrees and led to blurred or incorrect segmentation results.

Kumar et al. (2020) employed the Fuzzy C Means clustering (FCM) technique for segmenting cancerous regions in dermoscopic images. Subsequently, the segmented images were transformed into vectors utilizing two global descriptors, namely the Gray Level Co-Occurrence Matrix (GLCM) and the Local Binary Pattern (LBP). Finally, cancer types were classified by implementing an Artificial Neural Network (ANN) model with the differential evolution algorithm. In medical applications, ANN was sensitive to lighting conditions, noise, variations in medical image quality, and other factors.

Murugan et al. (2019) applied the watershed segmentation technique to delineate non-cancerous and cancerous regions in dermoscopic images. Furthermore, feature extraction was carried out utilizing the GLCM descriptor and the ABCD rule. The vectors obtained from the GLCM descriptor and ABCD rule were passed into Support Vector Machine (SVM), random forest, and K Nearest Neighbor (KNN). Among these classification models, SVM yielded superior classification results. However, SVM exhibits three significant issues in medical image classification: i) sensitivity to outliers and noise, ii) limited flexibility, and iii) limited scalability.

Toğaçar et al. (2021) initially employed an autoencoder model for reconstructing the collected ISIC dataset. Then, the structured and original datasets were classified implementing the MobileNetV2 model, which comprises spiking networks and residual blocks. However, the MobileNetV2 model exhibits three issues in disease detection: i) limited contextual understanding, ii) poor trade-off between accuracy and speed, and iii) difficulty in managing class imbalance.

Serte and Demirel (2019) designed a Gabor wavelet based CNN model to achieve accurate detection of seborrheic keratosis and malignant melanoma. Initially, the model decomposed input dermoscopic images into seven sub-bands, which were subsequently fed into eight parallel CNNs for skin lesion classification. The developed Gabor wavelet based CNN model was efficient in disease detection, but was computationally costly. Arora et al. (2022) integrated speeded up robust features with the quadratic SVM for skin cancer detection. However, the quadratic SVM comprises the following issues in skin cancer detection: i) poor interpolation between classes, ii) risk of overfitting, and iii) computational complexity.

Amin et al. (2020) initially resized dermoscopic images to 240×240×3 dimensions. The Otsu thresholding algorithm was then integrated with the bi-orthogonal two dimensional wavelet transformation technique for skin lesion segmentation. Pre-trained deep learning models, specifically Visual Geometry Group (VGG)-16 and AlexNet, were applied for feature extraction. Finally, Principal Component Analysis (PCA) and various machine-learning classification models were applied for feature dimensionality reduction and skin cancer detection. As observed in this literature, the pre-trained models extracted correlated and redundant features, leading to ineffective model training.

Thurnhofer-Hemsi et al. (2021) implemented a DenseNet201 model for precise detection of MSC. Additionally, Ali et al. (2021) developed a deep CNN model based on transfer learning for classifying malignant and benign skin lesions. In the developed deep CNN model, firstly, a kernel or filter was applied for eliminating artifacts and noise from dermoscopic images. Secondly, the denoised images were normalized and extracted discriminative features for precise image classification. The developed deep CNN model’s performance was compared with a few pre-trained CNN models, namely MobileNet, DenseNet, VGG-16. ResNet and AlexNet. The deep CNN model achieved higher classification results on the HAM10000 dataset, but was computationally expensive.

Sayed et al. (2021) have integrated the CNN model (Squeeze-Net) with the bald eagle search optimization algorithm for melanoma prediction. Similarly, in the works of Zhou and Arandian (2021), Tan et al. (2019), and Mohakud and Dash (2022b), the wildebeest herd optimization algorithm, improved particle swarm optimization algorithm, and grey wolf optimization algorithm were integrated with the CNN model for precise classification of malignant and benign skin lesions. Generally, the integration of an optimization algorithm with the CNN model increases resource requirements and training time.

Chaturvedi et al. (2020) conducted multiclass skin cancer detection utilizing five different CNN models, namely NASNet-large, Xception, Inception-ResnetV2, InceptionV3, and ResNeXt101 on the HAM10000 dataset. Among these models, the ResNeXt101 model was efficient in MSC classification, because it was attributed to its optimized architecture and has better capability in gaining high classification accuracy. Rashid et al. (2022) have employed the MobileNetV2 model for melanoma classification. Several image augmentation methods were used for tackling the class imbalance problem. The efficiency of the MobileNetV2 model was validated on the ISIC 2020 dataset. Additionally, Kaur et al. (Kaur et al.) employed a less complex and light-weighted CNN model for superior classification of MSC. The developed model’s performance was tested on different dermoscopic images, which were acquired from ISIC 2020, 2017, and 2016 datasets. Five different evaluation metrics were used for analyzing the efficacy of the developed CNN model. As discussed in earlier literature, CNN models often entail high computational costs. In order to highlight the above-mentioned problems and to achieve better MSC detection, a novel deep learning based automated framework is introduced in this manuscript.

## 3 Methods

**FIGURE 1:**
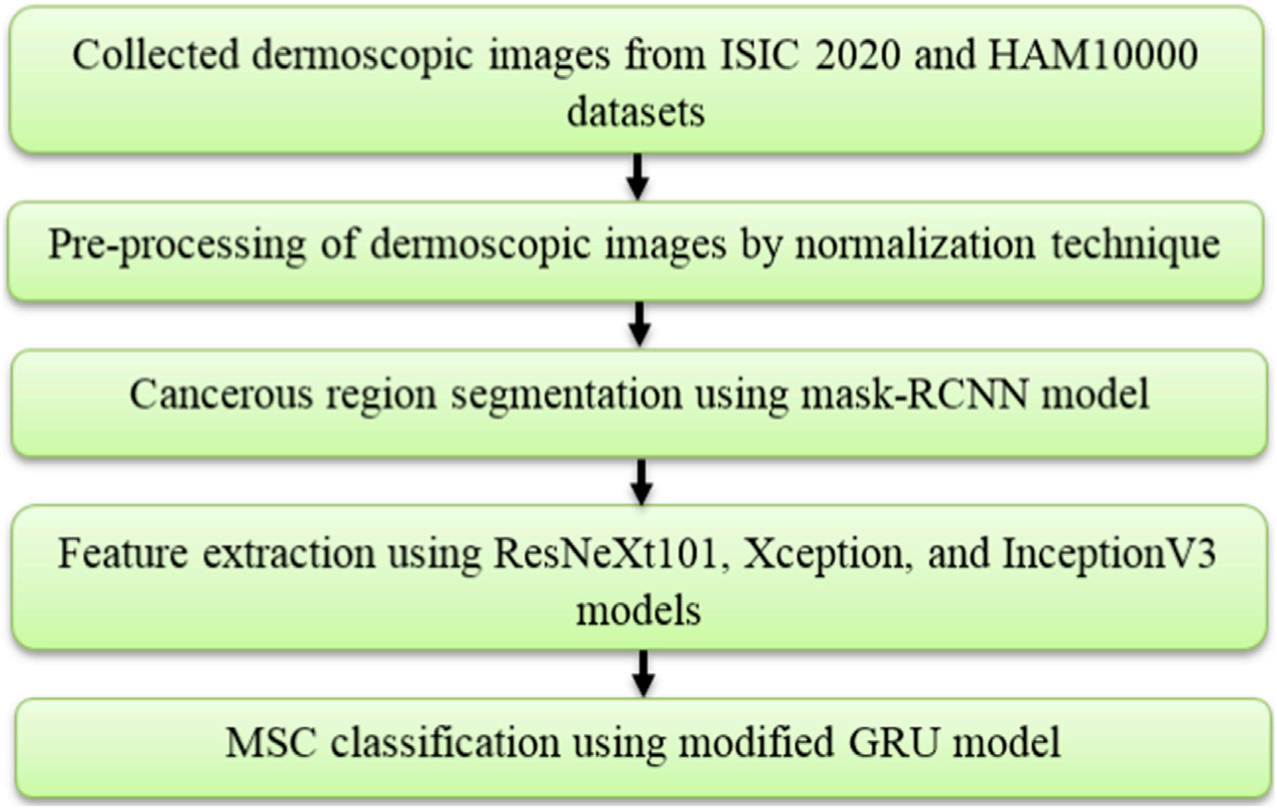
Design of the proposed framework.

In the application of MSC detection, the introduced deep learning based automated framework comprises five steps, namely **image collection:** ISIC 2020 and HAM10000 datasets, **image pre-processing:** normalization technique, cancerous region segmentation: mask-RCNN model, feature extraction: ResNeXt101, Xception, and InceptionV3 models and **MSC classification:** modified GRU model. The process involved in this framework is shown in Figure 1.

### 3.1 Dataset description

The mask-RCNN and modified GRU model’s performance are tested using two online benchmark datasets, namely ISIC 2020 dataset and HAM10000 dataset.

#### 3.1.1 ISIC 2020 dataset

**FIGURE 2:**
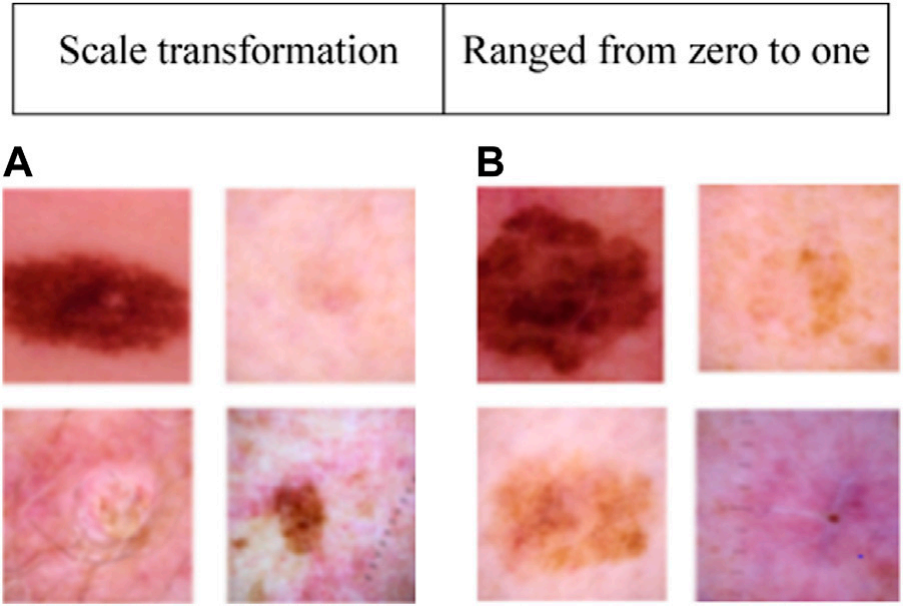
Sample images of the ISIC 2020 dataset: **(A)** Benign class, and **(B)** Malignant class.

This dataset consists of 33,126 dermoscopic images, with 32,542 representing benign lesions and 584 depicting malignant lesions. These 33,126 dermoscopic images were acquired from 2,000 distinct patients. In the ISIC 2020 dataset, 584 melanoma images and 11,670 benign class images are used for numerical examination. To manage the class imbalance problem, 4,522 melanoma images from the ISIC 2019 dataset are combined with the 584 melanoma images from the ISIC 2020 dataset (Rotemberg et al., 2021). Furthermore, several image augmentation methods are employed to augment the training dataset, namely shear transformation, horizontal flip, zoom transformation, rotation transformation, and scale transformation. The settings of these image augmentation methods are given in Table 1. Collectively, these methods generate approximately 6,564 augmented melanoma images. The sample images of the ISIC 2020 dataset are presented in Figure 2.

**TABLE 1:** Image augmentation methods with their setting.

Methods	Setting
Shear transformation	20o
Horizontal flip	True
Zoom transformation	0.20
Rotation transformation	25o
Scale transformation	Ranged from zero to one

#### 3.1.2 HAM10000 dataset

**FIGURE 3:**
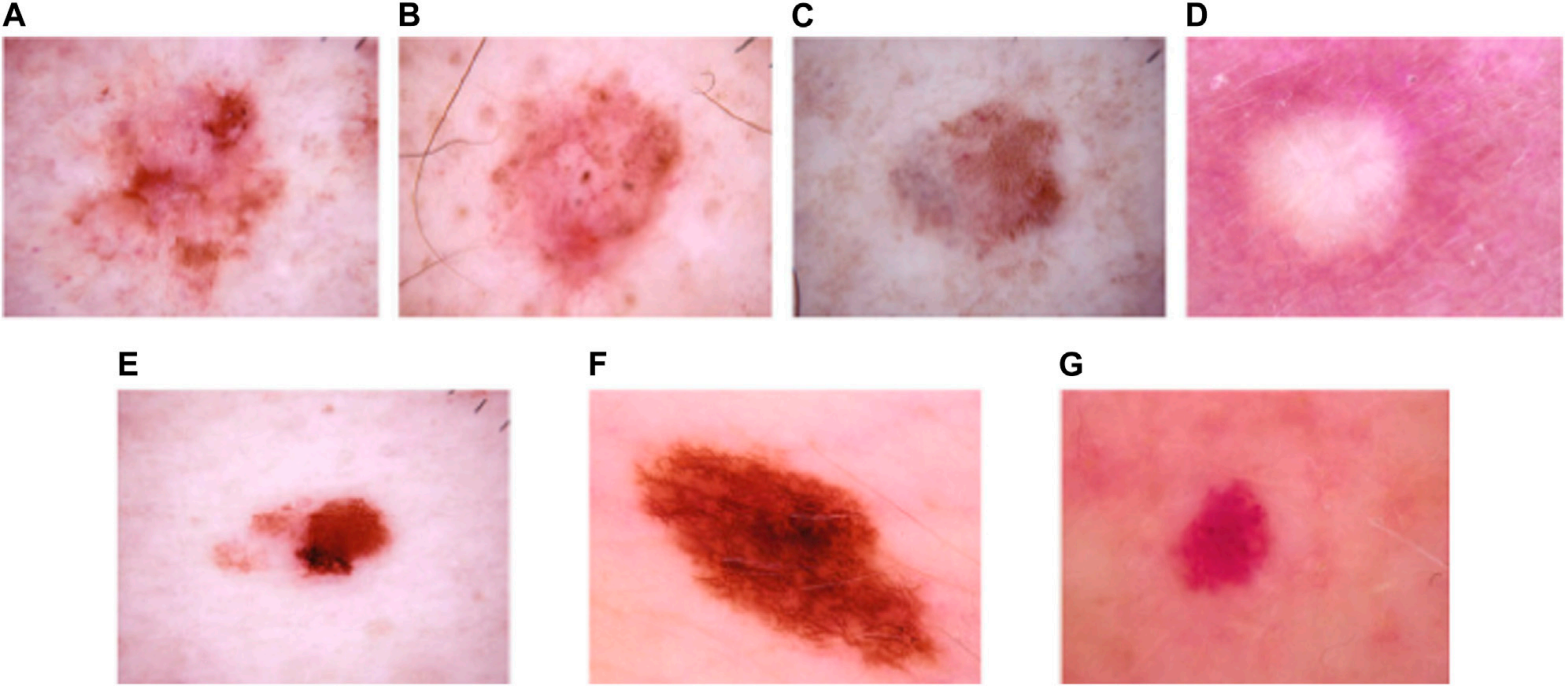
Sample images of the HAM10000 dataset: **(A)** AKIEC, **(B)** BCC, **(C)** BKL, **(D)** DF, **(E)** MEL, **(F)** NV, and **(G)** VASC.

It is one of the extensively utilized publicly available datasets for MSC detection (Tschandl et al., 2018). This dataset consists of 10,015 dermoscopic images belonging to seven classes, namely: Vascular Skin (VASC), Melanoma (MEL), Nevi (NV), Benign Keratosis (BKL), Actinic Keratosis (AKIEC), Dermatofibroma (DF), and Basal Cell Carcinoma (BCC). The number of dermoscopic images in the seven skin cancer types are presented as follows: VASC (142 samples), MEL (1113 samples), NV (6705 samples), BKL (1099 images), AKIEC (327 samples), DF (115 samples), and BCC (514 samples). The sample dermoscopic images of the HAM10000 dataset is shown in Figure 3.

### 3.2 Image pre-processing

After acquiring dermoscopic images from the ISIC 2020 dataset and HAM10000 dataset, image pre-processing is conducted using a normalization technique (Zhu et al., 2020). The acquired dermoscopic images are resized to 256×256 , where this process dramatically enhances the proposed model’s performance by speeding up the training process. In this context, a normalization technique is employed for eliminating data duplicacy. Initially, the dermoscopic images $I\left(x,y\right)$ are transformed into grayscale images $I^{\prime }\left(x,y\right)$ , and further, its histogram value is computed, as mentioned in Eq. 1.

Subsequently, the mean value of every dermoscopic image is calculated utilizing the average function, and it is mathematically expressed in Eq. 2. Furthermore, the co-relation between dermoscopic images is computed utilizing Eq. 3. When the co-relation between two dermoscopic images is higher than 0.99, the similar/identical image is eliminated, and lastly, the transformed grayscale images are converted to color dermoscopic images.

$$h1=\mathrm{histogram}\left(I\left(x,y\right)\right),h2=\mathrm{histogram}\left(I^{\prime }\left(x,y\right)\right)$$ (1)

$$h1=\mathrm{mean}\left(h1\right),h2=\mathrm{mean}\left(h2\right)$$ (2)

$$Co-relation=\frac{\sum_{x}\sum_{y}\left(I\left(x,y\right)-h1\right)\left(I^{\prime }\left(x,y\right)-h2\right)}{\sqrt{\left(\sum_{x}\sum_{y}{\left(I\left(x,y\right)-h1\right)}^{2}\right)}\left({\sum_{x}\sum_{y}\left(I^{\prime }\left(x,y\right)-h2\right)}^{2}\right)}$$ (3)

### 3.3 Cancerous region segmentation

After image pre-processing, the cancerous regions are precisely segmented by implementing the mask-RCNN model. The mask-RCNN model is an effective deep learning model implemented for instance segmentation and object detection tasks in computer vision applications, such as skin lesion detection (Wang et al., 2021). The mask RCNN model is an updated version of the faster-RCNN model, which is designed to perform pixel-level segmentation with object localization. In the context of MSC detection, the mask-RCNN model efficiently delineates and identifies various skin regions (skin anomalies, melanomas, and moles) in dermoscopic images. This model works by detecting bounding boxes around the skin lesions utilizing the Region Proposal Network (RPN) component (Su et al., 2021). Furthermore, it refines these bounding boxes and creates segmentation masks, which accurately outline the boundaries of every lesion. The mask-RCNN comprises three major components in MSC detection, which are briefly explained below;

• **Backbone network:** This model extracts hierarchical features from pre-processed dermoscopic images using a CNN model called ResNet.
• **RPN:** The RPN identifies potentially interesting regions (areas containing skin lesions) within pre-processed dermoscopic images. The selected proposals are then further refined in subsequent steps.
• **Mask head and Region of Interest (RoI) alignment:** RoI alignment is employed to pool regions of interest for generating feature maps with fixed size. Subsequently, the selected regions are processed by a mask head to predict pixel-wise segmentation masks for every proposed skin lesion.

**FIGURE 4:**
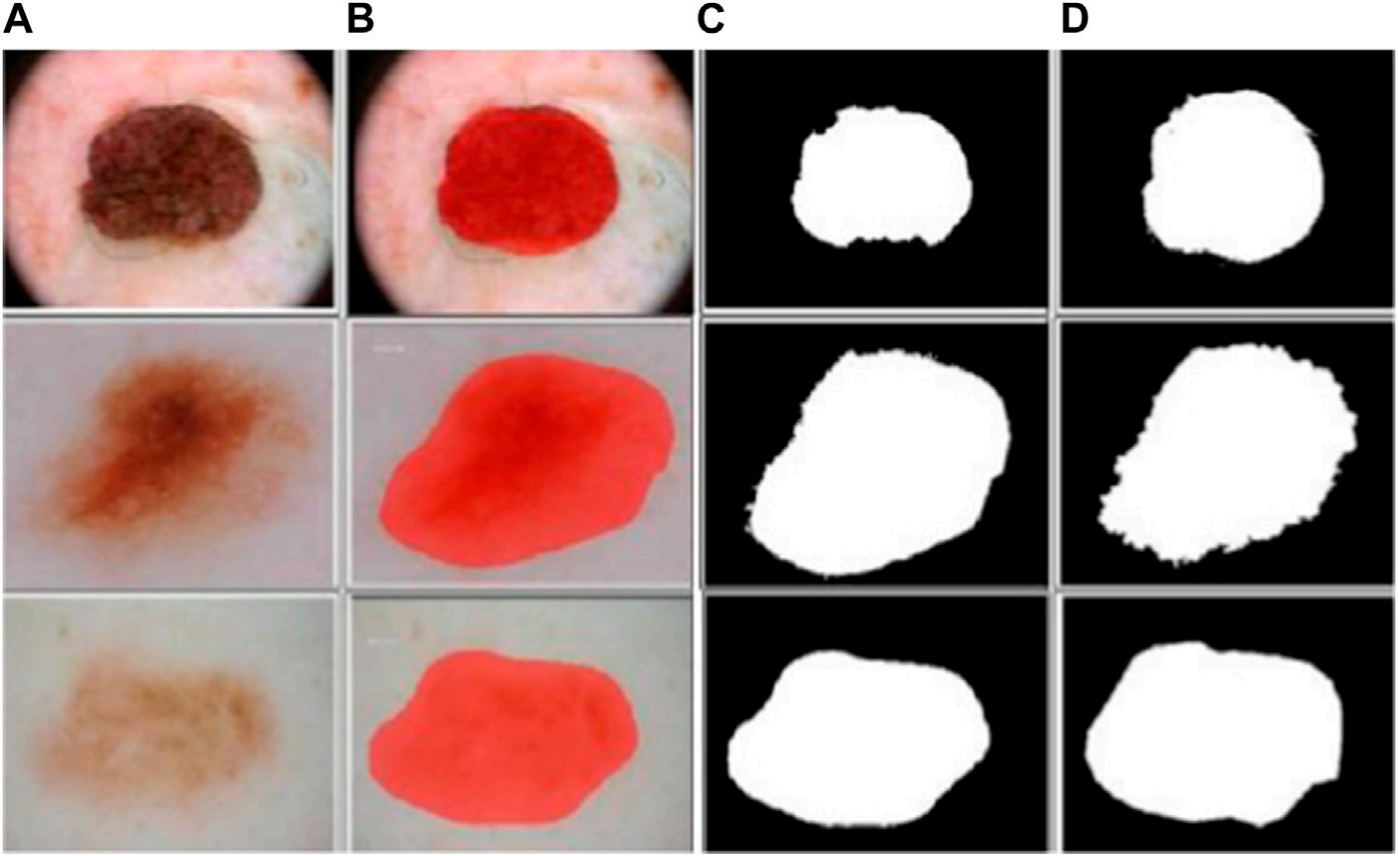
Sample segmented dermoscopic image: **(A)** pre-processed images, **(B)** output images of mask-RCNN model, **(C)** binary images of mask-RCNN model, and **(D)** ground-truth images.

In the context of MSC detection, the mask-RCNN model is trained utilizing annotated skin lesion images. These images are annotated with both pixel-level mask annotations and bounding box annotations. The mask-RCNN model then optimizes the parameters of several components for precisely detecting and segmenting skin lesions in unseen dermoscopic images. The sample segmented dermoscopic images are shown in Figure 4.

### 3.4 Feature extraction

After segmenting the cancerous regions using the mask-RCNN model, feature extraction is carried-out employing three pre-trained CNN models: ResNeXt101, Xception, and InceptionV3. These pre-trained CNN models transform pixel data from dermoscopic images into sets of meaningful and relevant feature vectors. This process reduces the framework’s complexity and addresses the “curse of dimensionality” problem caused by increased memory requirements and computational inefficiency. The theoretical explanation about the pre-trained CNN models: ResNeXt101, Xception, and InceptionV3 are presented below;

#### 3.4.1 ResNeXt101

The ResNeXt101 model efficiently captures hierarchical and complex patterns, and learns intricate high-level and low-level features from segmented dermoscopic images to achieve accurate MSC classification (Karanam et al., 2022). The ResNeXt101 model includes dense layers with ReLU activation function, Softmax, and dropout layers. The assumed parameters of the ResNeXt101 model are, learning rate is 0.0001, epochs is 100, momentum is 0.9, and optimizer is Stochastic Gradient Descent (SGD).

#### 3.4.2 Xception

Xception is a depthwise separable CNN model, which captures complex relationships and fine details in dermoscopic images. It includes regularization techniques: depthwise separable convolutions and batch normalization to overcome overfitting problems (Salim et al., 2023). Xception learns high dimensional feature vectors (global and local patterns) in dermoscopic images, which play a crucial role in skin cancer detection. The assumed parameters for the Xception model include a learning rate of 0.001, 100 epochs, and the Adam optimizer.

#### 3.4.3 InceptionV3

**FIGURE 5:**
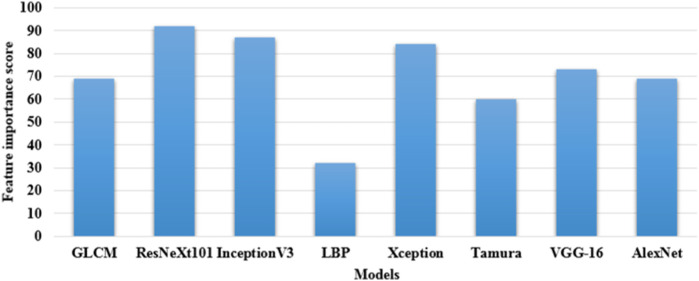
Calculation of feature importance score.

This architecture employs a series of convolutions with varying filter sizes for extracting feature vectors. InceptionV3 efficiently optimizes the trade-off between performance and computation by leveraging different kernel sizes. This model is fine-tuned using a learning rate of 0.001, the optimizer of Adam, a momentum of 0.9, and trained for 100 epochs (Ramaneswaran et al., 2021). These three pre-trained CNN models: ResNeXt101, Xception, and InceptionV3 extracts nearly 7,820 and 8,320 feature vectors from the ISIC 2020 dataset and HAM10000 dataset, respectively. In this scenario, these three feature extraction models are selected by computing feature importance score, which is shown in Figure 5. By inspecting Figure 5, in comparison to the ResNeXt101, Xception, and InceptionV3 models, the existing models: GLCM, LBP, Tamura, AlexNet, and VGG-16 have minimal feature importance score on the ISIC 2020 dataset and HAM10000 dataset.

### 3.5 MSC classification

The extracted 7,820 and 8,320 feature vectors from the ISIC 2020 dataset and HAM10000 dataset are fed into the modified GRU model for dermoscopic image classification. The conventional GRU model is a type of RNN, which utilizes a gating process for controlling the information flow in the network (Huang et al., 2020). The conventional GRU model comprises two gates (update and reset gates) that regulate the information retention and update process. These gates also assist in remembering and capturing relevant patterns in the extracted feature vectors (Venkataramaia et al., 2020; Li et al., 2021).

In the modified GRU model, the traditional activation functions, namely hyperbolic tangent and sigmoid are replaced with the swish-ReLU activation function. This replacement offers certain benefits due to its improved gradient flow and smoothness. During data training, the swish-ReLU activation function mitigates problems related to vanishing gradients that offer more efficient and stable learning. Additionally, the improved gradient flow enhances training stability, prevents neurons from becoming completely inactive, and accelerates the convergence rate. The swish-ReLU activation function potentially decreases the number of iterations required to achieve a certain level of accuracy in dermoscopic image classification. It also provides a mild form of regularization that reduces the risks of overfitting within the network.

Initially, the GRU model modulates the extracted feature vectors into units without utilizing a memory cell. In this context, the swish-ReLU activation function linearly interpolates between the prior and current candidate functions, as mathematically specified in Eq. 4.

$$h_{t}^{j}=\left(1-z_{t}^{j}\right){\;h}_{t-1}^{j}+z_{t}^{j}{\tilde{h}}_{t}^{j}$$ (4)

Where, ${\tilde{h}}_{t}^{j}$ denotes the current candidate function, as defined in Eq. 5. Additionally, the variable t stands for time, ${\;h}_{t-1}^{j}$ specifies the prior candidate function of the modified GRU model, and $h_{t}^{j}$ represents the activation function of the modified GRU model. Moreover, the update gate $z_{t}^{j}$ within the modified GRU model determines the extent to which the unit needs to modify its activation function. The mathematical formulation of the updated gate $z_{t}^{j}$ is provided in Eq. 6.

 h∼tj=swish wAt+Urrtj×ht−1j (5)

$$z_{t}^{j}=swish{\left(w_{z}A_{t}+U_{z}h_{t-1}\right)}^{j}$$ (6)

Whereas, $r_{t}^{j}$ indicates reset gate, and its mathematical formulation is given in Eq. 7.

$$r_{t}^{j}=swish{\left(w_{r}A_{t}+h_{t-1}\right)}^{j}$$ (7)

Where, swish represents the swish-ReLU activation function, w indicates a weight parameter, and U indicates a SGD optimizer. The SGD optimizer U updates the weight parameter w using the gradient function $\frac{\partial L}{\partial w}$ with a learning rate α of 0.001. Additionally, the modified reset gate is expressed in Eq. 8, where, $\partial L/\partial w_{r}$ indicates the gradient loss function, $A_{t}$ denotes the extracted feature vectors, and $w_{r+1}=w_{r}-\alpha \partial L/\partial w_{r}$ .

$$r_{t}^{j}=swish{\left(w_{r+1}A_{t}+h_{t-1}\right)}^{j}$$ (8)

The assumed parameters of the modified GRU model are as follows: batch size is 64, epochs is 100, dropout rate is 0.5, and decay rate is 0.9. The numerical examination of the proposed model is detailed in Section 4.

## 4 Results

The mask-RCNN and modified GRU model’s efficiency is simulated utilizing the Matlab 2020a software, and the experimental investigation is conducted on a computer equipped with an Intel Core i7 multi-core processor, NVIDIA GeForce RTX 4080 graphics card, and 16 GB memory. The mask-RCNN and modified GRU model’s performance is analyzed utilizing seven different performance metrics, namely: Jaccard score, Dice score, MCC, accuracy, sensitivity, f1-score, and specificity on the ISIC 2020^1^ and HAM10000^2^ datasets. Additionally, the modified GRU model’s performance is validated with 20%:80% of data testing and training.

### 4.1 Performance metrics

The Jaccard score estimates the ratio of the ground-truth mask B to the intersection of the segmented mask A . The Jaccard score ranges between zero to one, where zero represents no overlap and one states an overlap between the ground-truth and predicted masks. The Jaccard score is defined in Eq. 9, where, A∪B states the union (image pixels are encompassed by both ground-truth and segmented masks) and A∩B represents the intersection (image pixels agreed by both masks). Correspondingly, the Dice score estimates the similarity between the ground-truth mask and segmented mask by mask-RCNN model, and its formula is given in Eq. 10, where, $\left|A\right|$ indicates the image pixels in the segmented mask and $\left|B\right|$ denotes the image pixels in the ground-truth mask.

$$J\left(A,B\right)=\frac{\left|A\cap B\right|}{\left|A\cup B\right|}$$ (9)

$$D\left(A,B\right)=\frac{2\times \left|A\cap B\right|}{\left|A\right|+\left|B\right|}$$ (10)

The performance metrics: MCC, accuracy, sensitivity, f1-score, and specificity are commonly utilized for evaluating the efficacy of the classification model that is the modified GRU. These performance metrics are closely related to the information obtained from a confusion matrix. A confusion matrix is a table, which visualizes the effectiveness of a classification model by summarizing the number of False Negative (FN), False Positive (FP), True Negative (TN), and True Positive (TP) predictions.

The MCC accounts for all four confusion matrix values (FN, FP, TN, and TP). MCC provides a balanced result, even when the classes are imbalanced in the ISIC 2020 and HAM10000 datasets. Accuracy is a ratio of the total predictions to the number of correct predictions. The mathematical formulas of MCC and accuracy are denoted in Eqs 11, 12.

$$MCC=\frac{TP\times TN-FP\times FN}{\sqrt{\left(TP+FP\right)\left(TP+FN\right)\left(TN+FP\right)\left(TN+FN\right)}}\times 100$$ (11)

$$Accuracy=\frac{TP+TN}{TP+TN+FP+FN}\times 100$$ (12)

Sensitivity estimates the proportion of correctly predicted positive cases to the actual positive cases. F1-score is a harmonic mean of sensitivity and precision values. Specificity estimates the proportion of correctly predicted negative cases to the actual negative cases. The formulas utilized to compute sensitivity, f1-score, and specificity are represented in Eqs 13–15.

$$Sensitivity=\frac{TP}{TP+FN}\times 100$$ (13)

$$F1-score=\frac{2TP}{FP+2TP+FN}\times 100$$ (14)

$$Specificity=\frac{TN}{TN+FP}\times 100$$ (15)

### 4.2 Segmentation analysis

In this context, the numerical results of various segmentation models (K-means, FCM, FKM, superpixel clustering, Otsu thresholding, and mask-RCNN) are presented in Table 2. The segmentation model’s results are evaluated using two different performance metrics, namely Jaccard score and Dice score. As described in Table 2, the mask-RCNN model achieved 0.96 and 0.97 of Jaccard score and Dice score on the ISIC 2020 dataset. Similarly, the mask-RCNN model obtained 0.97 and 0.98 of Jaccard score and Dice score on the HAM10000 dataset. The obtained numerical outcomes are superior to the comparative models such as K-means, FCM, FKM, Superpixel, and Otsu thresholding. The mask-RCNN model adeptly handles several object orientations, shapes, and sizes within similar dermoscopic images. Therefore, it is more effective in scenarios where objects exhibit diverse appearances. The pixel-wise segmentation performed by the mask-RCNN model extracts rich semantic information, enabling more in-depth analysis in MSC detection.

**TABLE 2:** Numerical results of various segmentation models

Models	ISIC 2020 dataset		HAM10000 dataset	
	Jaccard score	Dice score	Jaccard score	Dice score
K-means	0.73	0.69	0.70	0.72
FCM	0.74	0.72	0.72	0.82
FKM	0.82	0.85	0.85	0.87
Superpixel	0.84	0.92	0.87	0.89
Otsu thresholding	0.90	0.94	0.89	0.92
Mask-RCNN	0.96	0.97	0.97	0.98

### 4.3 Classification analysis

The numerical results of various classification models on both ISIC 2020 and HAM10000 datasets are depicted in Table 3. The proposed classification model’s results are compared with other models such as RNN, ANN, Long Short Term Memory (LSTM) network, and GRU. By reviewing Table 3, it is evident that the modified GRU model has obtained impressive classification outcomes on both the ISIC 2020 and HAM10000 datasets. Specifically, the modified GRU model attained 99.84% and 99.88% of MCC, 99.95% and 99.98% of accuracy, 99.82% and 99.97% of sensitivity, 99.86% and 99.90% of f1-score, and 99.94% and 99.87% of specificity on the ISIC 2020 and HAM10000 datasets, respectively. These obtained results are superior to the existing classification models, namely RNN, ANN, LSTM, and GRU.

**TABLE 3:** Numerical results of various classification models.

ISIC 2020 dataset					
Models	MCC (%)	Accuracy (%)	Sensitivity (%)	F1-score (%)	Specificity (%)
RNN	93.80	94.38	94.66	96.48	96.80
ANN	96.72	96.82	96.79	97.42	97.60
LSTM	98.76	98.88	97.97	97.90	98.83
GRU	99.58	99.22	98.98	99.13	98.58
Modified GRU	99.84	99.95	99.82	99.86	99.94
HAM10000 dataset					
RNN	95.30	96.43	97.20	97.67	97.43
ANN	97.95	97.90	97.94	98.66	98.19
LSTM	98.66	98.46	98.78	98.80	98.60
GRU	99.12	99.28	98.86	99.12	99.25
Modified GRU	99.88	99.98	99.97	99.90	99.87

In the application of MSC detection, the modified GRU model has the potential to capture spatial and temporal patterns in dermoscopic images that helps in achieving high classification results. Additionally, the proposed modified GRU model has a deep understanding of both image processing techniques and RNN architectures that reduces overfitting and vanishing gradient problems with faster convergence. Furthermore, the efficacy of the modified GRU model is analyzed utilizing various K-folds on ISIC 2020 and HAM10000 datasets. The results of K-fold cross validation is mentioned in Table 4. As stated in Table 4, the modified GRU model achieved an efficient result in MSC detection, particularly in 5-fold (20%:80% of testing and training) related to other types such as 2-fold (50%:50% of testing and training), 4-fold (25%:75% of testing and training), and 8-fold (12.50%:87.50% of testing and training). In the context of MSC detection, performing K-fold cross validation effectively mitigates overfitting and overcomes class imbalance problems.

**TABLE 4:** Results of K-fold cross validation.

Dataset	Measures (%)	K = 2	K = 4	K = 5	K = 8
ISIC 2020	MCC	96.76	96.80	99.84	98.20
	Accuracy	97.66	97.68	99.95	97.68
	Sensitivity	97.85	98.06	99.82	97.44
	F1-score	98.30	97.42	99.86	97.32
	Specificity	97.79	98.10	99.94	98.50
HAM10000	MCC	97.12	98.12	99.88	98.34
	Accuracy	98.98	97.87	99.98	98.09
	Sensitivity	96.45	97.45	99.97	98.12
	F1-score	97.80	98.05	99.90	97.96
	Specificity	98.73	98.33	99.87	98.22

### 4.4 Comparative analysis

The proposed modified GRU model’s effectiveness is compared with existing models developed by Thurnhofer-Hemsi et al. (2021), Ali et al. (2021), Chaturvedi et al. (2020), Rashid et al. (2022), and Kaur et al. (Kaur et al.). Thurnhofer-Hemsi et al. (2021) integrated transfer learning with five CNN models (MobileNetV2, InceptionV3, GoogleNet, Inception-ResNetV2, and DenseNet201) for precise detection of skin cancer. Empirical analysis confirmed that the DenseNet201 model achieved a high classification accuracy of 95% on the HAM10000 dataset. Ali et al. (2021) employed a deep CNN model for precise classification of malignant and benign skin lesions. Compared to conventional pre-trained models, the deep CNN model obtained a testing accuracy of 91.93%. Additionally, Chaturvedi et al. (2020) performed skin cancer detection using various pre-trained CNN models, including Xception, NASNet-large, InceptionV3, Inception-ResnetV2, and ResNeXt101. Empirical analysis revealed that the ResNeXt101 model achieved a high accuracy of 93.20%. In comparison to these aforementioned studies, the proposed modified GRU model achieved an exceptional accuracy of 99.98% on the HAM10000 dataset, as depicted in Table 5.

**TABLE 5:** Results comparison on the HAM10000 dataset.

Models	Dataset	Classification accuracy (%)
DenseNet201 (Thurnhofer-Hemsi et al., 2021)	HAM10000 (7 classes)	95
Deep CNN (Ali et al., 2021)		91.93
ResNeXt101 (Chaturvedi et al., 2020)		93.20
Modified GRU		99.98

Rashid et al. (2022) employed the MobileNetV2 model for the classification of benign and melanoma skin lesions on the ISIC 2020 dataset. Numerical examination reveals that the MobileNetV2 model achieved an accuracy of 98.20% on the ISIC 2020 dataset. Similarly, Kaur et al. (Kaur et al.) developed a light-weighted CNN model for superior classification of benign and melanoma skin lesions on the ISIC 2020 dataset. The results indicate that the developed light-weighted CNN model performed efficiently on balanced and large skin cancer datasets like ISIC 2020. In this context, the light-weighted CNN model achieved a classification accuracy of 90.48% on the ISIC 2020 dataset. In comparison to these existing models, the proposed modified GRU model achieved an exceptional accuracy of 99.95% on the ISIC 2020 dataset, as mentioned in Table 6.

**TABLE 6:** Results comparison on the ISIC 2020 dataset.

Models	Dataset	Classification accuracy (%)
MobileNetV2 (Rashid et al., 2022)	ISIC 2020 (2 classes)	98.20
Light-weighted CNN (Kaur et al.)		90.48
Modified GRU		99.95

### 4.5 Discussion

The precise segmentation and classification of skin lesions are crucial aspects of this research. The primary benefit of utilizing the mask-RCNN model in image segmentation is precise instance segmentation. Conventional semantic segmentation groups image pixels into categories, whereas the mask RCNN model outlines and differentiates individual object instances in dermoscopic images. This mechanism leads to more detailed and accurate segmentation results, which are vital in tasks like MSC detection. Additionally, as discussed in the quantitative section, the modified GRU model is more efficient in dermoscopic image classification compared to other classification models. The modified GRU model effectively captures the temporal relationships and dependencies in dermoscopic images that results in enhanced classification performance. Moreover, both the mask-RCNN model and modified GRU model consumes minimal computational time during segmentation and classification, as depicted in Tables 7, 8.

**TABLE 7:** Computational time for region segmentation.

Computational time for segmentation (seconds)		
Models	ISIC 2020 dataset	HAM10000 dataset
K-means	12.02	16.43
FCM	13.24	16.10
FKM	10.53	12.35
Superpixel	8.51	9.26
Otsu thresholding	9.22	10.21
Mask-RCNN	6.20	7.25

**TABLE 8:** Computational time for image classification.

Computational time for image classification (seconds)		
Models	ISIC 2020 dataset	HAM10000 dataset
RNN	10	12.04
ANN	11.28	13.40
LSTM	9.44	9.32
GRU	7.50	8.36
Modified GRU	5.24	6.03

## 5 Conclusion

In the current scenario, early detection and prognosis of melanoma efficiently reduce the mortality rate and improve survival rates. The primary objective of this manuscript is to segment and classify lesion regions. The proposed framework relies on deep learning models for both segmentation (mask-RCNN model) and classification (modified GRU model) steps. Furthermore, three pre-trained models (ResNeXt101, Xception, and InceptionV3) are employed to extract relevant feature vectors from dermoscopic images. This process reduces unnecessary processing, rendering the proposed framework computationally efficient. Seven distinct performance metrics are utilized to analyze the efficiency of the proposed models (mask-RCNN and modified GRU). Empirical investigation demonstrates that the mask-RCNN model achieves more accurate segmentation results than existing models in light of Jaccard score and Dice score. Additionally, the modified GRU model achieves an impressive classification accuracy of 99.95% and 99.98% on the ISIC 2020 and HAM 10000 datasets with limited computational time. In future work, the proposed modified GRU model can be validated on an enormous dataset with more labeled skin lesions by including feature selection step in order to gain high classification accuracy.

## Data Availability

The original contributions presented in the study are included in the article/supplementary material, further inquiries can be directed to the corresponding author.

## Footnotes

1 ISIC 2020 dataset: https://challenge2020.isic-archive.com/

2 HAM10000 dataset: https://www.kaggle.com/datasets/kmader/skin-cancer-mnist-ham10000
